# The Binding Mode of the Sonic Hedgehog Inhibitor Robotnikinin, a Combined Docking and QM/MM MD Study

**DOI:** 10.3389/fchem.2017.00076

**Published:** 2017-10-23

**Authors:** Manuel Hitzenberger, Daniela Schuster, Thomas S. Hofer

**Affiliations:** ^1^Theoretical Chemistry Division, Institute of General, Inorganic and Theoretical Chemistry, University of Innsbruck, Innsbruck, Austria; ^2^Department of Physics, Theoretical Biophysics (T38), Technical University of Munich, Munich, Germany; ^3^Pharmaceutical Chemistry, Institute of Pharmacy, University of Innsbruck, Innsbruck, Austria

**Keywords:** sonic hedgehog (Shh), QM/MM, robotnikinin, sonic hedgehog inhibitor, metalloproteins, density functional theory, docking studies, molecular dynamics simulation

## Abstract

Erroneous activation of the Hedgehog pathway has been linked to a great amount of cancerous diseases and therefore a large number of studies aiming at its inhibition have been carried out. One leverage point for novel therapeutic strategies targeting the proteins involved, is the prevention of complex formation between the extracellular signaling protein Sonic Hedgehog and the transmembrane protein Patched 1. In 2009 robotnikinin, a small molecule capable of binding to and inhibiting the activity of Sonic Hedgehog has been identified, however in the absence of X-ray structures of the Sonic Hedgehog-robotnikinin complex, the binding mode of this inhibitor remains unknown. In order to aid with the identification of novel Sonic Hedgehog inhibitors, the presented investigation elucidates the binding mode of robotnikinin by performing an extensive docking study, including subsequent molecular mechanical as well as quantum mechanical/molecular mechanical molecular dynamics simulations. The attained configurations enabled the identification of a number of key protein-ligand interactions, aiding complex formation and providing stabilizing contributions to the binding of the ligand. The predicted structure of the Sonic Hedgehog-robotnikinin complex is provided via a PDB file as Supplementary Material and can be used for further reference.

## 1. Introduction

The Hedgehog (Hh) family of proteins derives its name from the malformations that occur to larvae of drosophila flies upon altering of the respective gene (Varjosalo and Taipale, 2008). While for drosophila and other invertebrates only one variant of this protein is known, at least three different forms occur in vertrebrates, namely Indian Hedgehog (Ihh), Desert Hedgehog (Dhh) and Sonic Hedgehog (Shh) (Ingham and McMahon, 2001; Varjosalo and Taipale, 2008). Incidentally, Dhh is more closely related to the drosophila variant of the protein, whereas Ihh and Shh share a lot of similarities, implying a more recent gene duplication event (Ingham and McMahon, 2001). The Hedgehog signaling pathway plays an important role in several crucial events during embryogenesis, including patterning of the neural tube, limb and lung development, it also steers the segmentation of insect bodies (Ingham and McMahon, 2001; Jeong and McMahon, 2004; Varjosalo and Taipale, 2008). With the exception of adult stem cell differentiation (Palma et al., 2004) Hh signaling is mostly dormant in adults, however, aberrant activation of the pathway has been linked to a large number of cancerous diseases (Hahn et al., 1996; Goodrich et al., 1997; Berman et al., 2003; Hamed et al., 2004; Kubo et al., 2004) like for example bladder cancer, medulloblastoma, breast cancer, esophageal cancer or rhabdomyosarcoma and therefore has increasingly been targeted as a leverage point for novel anti-cancer therapies.

Shh is the most widely (Varjosalo and Taipale, 2008) expressed Hedgehog variant in vertebrates and thus most information concerning the biochemical pathways involving Hedgehog proteins has been gathered through investigations of Shh (Hwang et al., 2011). Sonic Hedgehog is synthesized inside the cell as a 45 kDa precursory protein, consisting of a 20 kDa N-terminal signaling and a C-terminal auto-catalytically active processing domain. Upon cleavage of the C-terminus, the remaining signaling peptide is modified with an N-terminal palmitic acid moiety and a C-terminal cholesterol molecule in order to mature into the morphologically active ShhN form (also referred to as Shh from this point on). The processed protein is then secreted into the extracellular matrix, where it acts as a ligand for the transmembrane protein Patched 1 (Ptc1). The binding of Shh to Ptc1 mediates the release of another transmembrane protein, Smoothened (Smo) which in turn migrates to the cell's primary cilium from where it activates the glioma-associated oncogene (Gli) transcription factors, thereby promoting the expression of Shh pathway-specific genes. An alternative binding partner on the cellular surface is the Hedgehog-interacting protein (Hhip), which is upregulated upon Shh binding and functioning as a decoy for Shh, hence acting as an antagonist for pathway activation (Ingham and McMahon, 2001; Varjosalo and Taipale, 2008; Bosanac et al., 2009). Most approaches, aiming to counteract the abnormal activation of the Hh pathway are targeting the deactivation of Smo or the transcription factors themselves (Varjosalo and Taipale, 2008; De Smaele et al., 2012). Another viable approach would be the inhibition of Shh binding to Ptc1, thereby increasing the therapeutic selectivity by minimizing the risk of unwanted deactivation of important biological pathways that are associated with Smo or Gli but independent of Shh (Rimkus et al., 2016). Known potential ligands include antibodies (Maun et al., 2010) as well as the small molecule robotnikinin (Mol. weight = 454.95g/mol; Stanton et al., 2009).

According to various investigations conducting X-ray crystallography, Shh possesses three divalent metal ions: Two Ca(II) ions, bound in loop regions by residues E90, E91, E127, D96, D130, and D132 (McLellan et al., 2008) and a Zn(II) ion, coordinated by two histidine residues (H141, H183), an aspartate (D148) and a water molecule, bridging the ion with glutamate E177 (Bishop et al., 1999, 2009; Bosanac et al., 2009). Structurally, the zinc site is analogous to those of zinc hydrolases such as thermolysin or bacterial carboxypeptidase A (Hall et al., 1995), however, extensive studies of Shh could not confirm any enzymatic activity (Fuse et al., 1999). Still, the existence of these ions suggests that they are important for Shh to carry out its role in the pathway, hence several experimental and theoretical studies have been undertaken to uncover their influence (Bishop et al., 1999, 2009; McLellan et al., 2008; Bosanac et al., 2009; Maun et al., 2010; Hwang et al., 2011, 2013).

This work is a follow-up on a recent computational study investigating the role of the metal ions of Shh, utilizing classical molecular mechanics (MM), as well as hybrid quantum mechanical(QM)/molecular mechanical molecular dynamics (MD) simulations (Hitzenberger and Hofer, 2016). One of the findings of this study was that simple MM based approaches are not sufficient to provide an accurate model for the complex interactions present in the Zn(II) binding site. The utilized DFT BP86-D3 (Perdew, 1986; Becke, 1988; Grimme et al., 2010), triple zeta (TZ) QM/MM (Warshel and Levitt, 1976; Lyne et al., 1987, 1990; Åqvist and Warshel, 1993) link bond (Hitzenberger and Hofer, 2015) approach, however, has been shown to be able to very accurately reproduce the available experimental data.

Targeting extracellular proteins, serving as ligands of transmembrane proteins can be a very challenging task which is highlighted by the effort required to discover robotnikinin necessitating the screening of a set of 10,000 diverse compounds (Stanton et al., 2009). Rational drug design could aid in the development of novel compounds that are able to inhibit Shh. To do this, however, requires the knowledge of the Shh-robotnikinin binding mode which is still unknown since no experimental data on this complex has been published yet. For this reason a QM/MM MD-refined docking study, providing detailed and highly accurate information on the interactions of robotnikinin with Shh is presented in this work. The combination of docking, force field approaches, quantum mechanics and molecular dynamics enables an exhaustive investigation of the system. By explicitly considering the dynamical aspects of the complex at QM level, the chosen approach is able to account for small conformational adaptations concerning the binding geometry and interaction profile (De Vivo et al., 2016).

## 2. Methodology

### 2.1. Classical simulation setup

The starting point for the docking of robotnikinin to Shh was the equilibrated classical simulation box, used in the previous investigations (Hitzenberger and Hofer, 2016), which are themselves based on an X-ray structure (Bosanac et al., 2009) (PDB:3HO5). All histidine residues were protonated at the ε-nitrogen, with the only exception being Hid 183, which was protonated at the δ-position to enable a binding geometry akin to the one predicted by X-ray investigations (Bosanac et al., 2009). The acidic and basic sidechains were all in the protonation state, predominately present at the physiological pH value. In order to generate a reasonable docking pose in which the ligand adequately occupies the binding groove, two iterations of docking with an intermittent MD simulation were necessary, since in the starting structures, stemming from simulations of empty Shh, the binding groove is not accessible to the ligand in its entirety. The first cycle of docking was performed using the software package MOE (Chemical Computing Group Inc., 2016), employing the AMBER-12:EHT force field and induced fit docking. The triangle matcher method was used to place the conformers of robotnikinin in the pseudo active site, while for scoring the London dG function was utilized prior to the refinement of the pose via the force field. After that, the poses were re-scored via the GVBI/WSA function, also used for the final ranking of the docking poses. Since all known interactions of Shh with its binding partners are mediated by amino acids present in the binding groove of Sonic Hedgehog (McLellan et al., 2008; Bosanac et al., 2009; Maun et al., 2010; Hwang et al., 2011, 2013), all residues in the respective region were selected as potential receptors in the docking step of the study. A set of diverse but highly ranked structures were selected for classical MD simulations in order to generate structures for the second docking cycle. The simulations were carried out using the AMBER-12SB (Zgarbova et al., 2011) force field in order to remain consistent with the settings used for docking. All ligand interactions were described by a GAFF force field generated via Antechamber (Case et al., 2014), a program part of the AMBER14 suite. Merz-Kollmann partial charges (Singh and Kollman, 1984) were derived by performing Hartree Fock (HF) calculations with a 6-31G^*^ (Hariharan and Pople, 1973; Krishnam et al., 1980; Francl et al., 1982; Clark et al., 1983; Gill et al., 1992) basis set using GAUSSIAN 09 (Frisch et al., 2009), as required by the AMBER force field. The Ca(II), Zn(II) ions of Shh as well as the chloride counter ions were described by the parameters (Aaqvist, 1990; Li and Merz, 2014) provided with the AMBER14 (Case et al., 2014) simulation package. The complexes were placed in periodic, cubic simulation boxes with a volume of ~540,000 Å^3^ and solvated in approximately 17,000 rigid TIP3P (Jorgensen et al., 1983) water molecules. The non-bonded cutoff was set to 10 Å and the long range interactions were treated by the particle mesh Ewald (PME) (Darden et al., 1993) method. In order to satisfy the requirements of the chosen NpT ensemble, temperature coupling was carried out via Langevin dynamics with a collision frequency of 1.0 ps^−1^, the pressure was controlled by the Berendsen manostat (Berendsen et al., 1984) with a relaxation time of 2 ps. The SHAKE (Ryckaert et al., 1977) algorithm was applied to constrain all bonds involving hydrogen, enabling a time step of 2.0 fs.

After an initial energy minimization of 60, 000 steps, utilizing the sander module of AMBER14 (Case et al., 2014), the systems were heated for 2 ns to the target temperature of 300 K using pmemd (Case et al., 2014) (MPI) and positional restraints to keep the protein-ligand complex fixed. Subsequently, the restraints were lifted and the systems equilibrated for 15 ns at a pressure of 1 atm, again using the MPI version of pmemd. The 100 ns production run was performed using the CUDA (Nickolls et al., 2008) implementation of pmemd, thereby considerably speeding up the process.

After the first MM MD run, the simulation in which the ligand displayed the lowest root mean square deviation was selected for the preparation of the actual simulation system by redocking the ligand using identical settings as before. The highest scoring structure was then used for another MM MD simulation, following the same protocol as above.

### 2.2. QM/MM setup

Choosing an appropriate QM method to describe the chemically most relevant part of the system is imperative to gain accurate and representative data from the simulation, therefore a reasonable compromise between accuracy and computational demand has to be made. DFT methods have been found to work fairly well when employed for systems containing metal ions (Kuta et al., 2006; Lepšík and Field, 2007; Hierao, 2011; Ryde and Grimme, 2011) and even though there are examples where DFT fails to deliver accurate results (Schwenk et al., 2004; Radoń and Pierloot, 2008; Yoo et al., 2009; Rowley and Roux, 2012; Gillan et al., 2016), at the moment it still represents the best tradeoff between computational cost and reliability (Senn and Thiel, 2009). Alternatives, like HF have been shown to be inadequate for such systems (Ryde and Grimme, 2011) and second-order Møller-Plesset perturbation theory (MP2), while computationally much more costly is known to occasionally perform worse than DFT (Ryde and Grimme, 2011). More sophisticated methods, such as Coupled Cluster (CC) or Configuration Interaction (CI) are too demanding to utilize them for the description of systems of the size studied in this work. Moreover, the previously published QM/MM study of this protein has shown that the used BP86 functional (Perdew, 1986; Becke, 1988), along with the cc-pVTZ (Dunning, 1989) (for C, H, N and O atoms) and def2-TZVP (Wiegend and Ahlrichs, 2005) (for Zn) basis sets and the D3 correction is able to adequately describe the system, while still being economical enough to enable acceptable trajectory lengths (Hitzenberger and Hofer, 2016). For this reason and in order to produce data that is directly comparable, the same setup has been chosen for this work. However, for one of the simulations, the double-zeta (DZ) versions of the mentioned basis sets have been used. The resolution of identity (RI) (Ren et al., 2012) approach has been employed alongside the D3 correction (Grimme et al., 2010) to speed up the calculation of the 4-center-2 electron integrals and to improve the description of dispersion effects, respectively.

The system has been partitioned into a QM and an MM zone with the focus of attention on the Zn(II) coordination site and the ligand, since the classical part of the investigation (docking and MM MD) strongly suggested that the Zn(II)-robotnikinin interaction is of great importance to the stability of the resulting complex. An energy and structure adjusted link atom approach was applied to describe all bonds penetrating the interface between the QM and the MM zone (Amara and Field, 2003; Lin and Truhlar, 2007; Hitzenberger and Hofer, 2015; Messner, 2015). In order to cleanly terminate the QM region without the introduction of artifacts stemming from the QM/MM coupling, a set of suitable link atom parameters {ρ, *r*_0_, *k*_*L*_} (Hitzenberger and Hofer, 2015) was derived and can be found in Table 1. The embedding of the QM into the MM zone was handled via the electrostatic embedding method, where the QM atoms interact with their MM counterparts via inclusion of MM partial charges into the QM Hamiltonian. While the charges of the QM atoms were updated at every step and calculated via the Mulliken method (Mulliken, 1962), the MM charges used were provided by the force field. The problem with such a setup is that MM point charges used in most popular force fields and water models are usually not tailored to accurately represent the electron density of a molecule but to reproduce certain observables (such as the permittivity of water) when applied together with the other force field parameters (Senn and Thiel, 2009). In general, it can be assumed that the point charges of the force field are not compatible with the DFT-derived Mulliken charges. The oxygen atom of a TIP3P water molecule (Jorgensen et al., 1983), for example, possesses a charge of −0.83*e*, while for a water molecule in bulk conditions where the partial charge is calculated by the used QM setup it is only −0.55*e*. Similar over-polarization can be witnessed with the amino acids of the protein. In order to minimize the possibility to sample artificially strong interactions between QM and MM species at the QM/MM interface, a (QM method dependent) scaling factor for all MM charges, used for embedding, has been applied. At the same time the charges used to calculate MM/MM interactions were not altered. The applied scaling factor is in principle a simplification of an embedding scheme where all charges next to a QM atom are described by a Gaussian distribution (Amara and Field, 2003), thereby providing a distance dependent charge scaling. This Gaussian scheme was confirmed to be a very accurate embedding method when compared to more traditional techniques (Amara and Field, 2003; König et al., 2005), however, it is not compatible with many popular QM programs and strongly dependent on the proper parametrization of the blurring width. Therefore, for this study, a fixed scaling factor of 0.666 was used for all QM/MM MD simulations as this ensured that the charges of water molecules in close proximity to the QM region are scaled down to −0.55*e*. Additionally, test calculations showed that the mean QM/MM charge deviation of the amino acids His, Asp, and Glu (all residues in very close proximity to the QM zone are of these types) is reduced from 0.17 to 0.08 as well when the scaling factor is applied. The use of this scaling factor already proved successful in a previous study of Shh (Hitzenberger and Hofer, 2016). All remaining interactions between the QM and MM species, like bending and torsional terms are handled via the force field. All terms, however, where the central atoms are exclusively QM species have been excluded in order to prevent extensive double counting of forces (Eurenius et al., 1996).

**Table 1:** Ideal link atom parameters for RI-BP86-D3, cc-pVTZ, or cc-pVDZ with embedding charges scaled by a factor of 0.666; ρ refers to the distance ratio between C_α_ and C_β_ on which the link atom is placed, *r*_0_ (in Å) and *k*_*L*_ (in kcal/mol/Å^2^) represent the minimum and the force constant of the harmonic energy correction potential of the link bond (Hitzenberger and Hofer, 2015), respectively.

**Link Bond**	**Triple Zeta Basis**			**Double Zeta Basis**		
	**ρ**	***r*_0_**	***k*_*L*_**	**ρ**	***r*_0_**	***k*_*L*_**
Hid^a^	0.7140	1.560	139.056	0.7226	1.564	143.739
Hie^b^	0.7155	1.561	145.779	0.7257	1.561	176.663
Glu	0.7241	1.547	170.580	0.7289	1.554	187.240
Asp	0.7251	1.543	145.051	0.7223	1.548	127.987
ROB 1^c^	0.7363	1.507	235.182	0.7435	1.514	242.270
ROB 2^d^	0.7332	1.515	240.882	0.7395	1.519	245.840

a *Histidine protonated at the δ position*.

b *Histidine protonated at the ε position*.

c *First robotnikinin link bond*.

d *Second robotnikinin link bond*.

**Figure 1:**
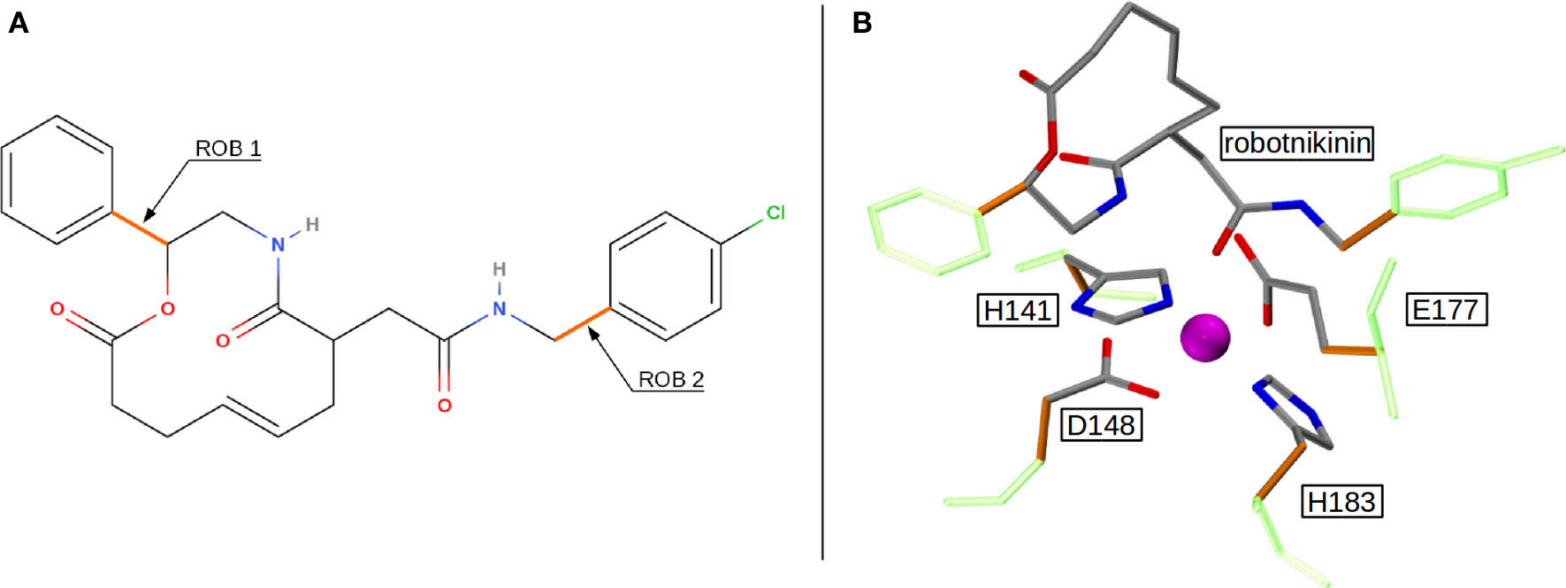
**(A)** QM/MM separation of robotnikinin. The macrocycle and the adjacent chain with the amide function are included in the QM zone, while the aromatic residues are excluded. The link bonds are depicted in orange. **(B)** The QM zone of the core simulation. Link bonds are colored in orange and the MM atoms of the QM/MM separated residues are depicted in light green, whereas the Zn(II) ion is shown in magenta.

Altogether, four different QM/MM MD simulations have been conducted in the course of this study. Table 2 presents an overview of the differences between the simulations. Since the MM derived data suggests that robotnikinin directly coordinates to the Zn(II) site, the QM zone for the first simulation (henceforth called “core simulation”) was chosen so that all important interactions around the ion were described by quantum mechanics. Therefore, the Zn(II) ion, the sidechains of H141, H183, D148, E177, the macrocycle, plus the chain containing the second amide function of robotnikinin (see Figures 1A,B for detailed information regarding QM/MM partitioning), as well as all water molecules within a radius of 5.5 Å were considered to be QM species. If robotnikinin as a whole would be included into the QM calculation then all residues in the vicinity of its phenyl and chlorophenyl rings would have to be described by QM as well because interactions between aromatics in the QM and the MM zone are very sensitive to the QM/MM potential and thus very difficult to describe correctly. Consequently, the addition of only the aromatic rings would have very likely lowered the predictive power of the simulation. The inclusion of all potential interaction partners of the rings in question would have lead to a very large QM system and thus making it impossible to sample a reasonable amount of configurations. In consequence, robotnikinin was truncated in the QM system and the substituents were described by the force field. Since the parametrization of the resulting link bonds was very thorough and the ionic site distant enough to justify an MM description of the aromatic moieties this provides an adequate compromise between effort and accuracy.

**Table 2:** Overview of the conducted QM/MM simulations.

**Simulation**	**System**	**QM atoms**	**Basis set**	**MD Steps/Simulation time**
#1 Core	Core	81	TZ	4,000/80 ps
#2 Extended (TZ)	Core+H134, H135	105	TZ	1,000/20 ps
#3 Extended (DZ)	Core+H134, H135	105	DZ	4,500/90 ps
#4 Empty	Empty Shh	42	DZ	6,000/120 ps

*The number of QM atoms includes hydrogen atoms but does not account for water molecules. The core system contains Zn(II), robotnikinin (excluding the aromatic rings), H141, D148, E177, and H183*.

The starting point of the core QM/MM simulation was the structure resulting from the final MM MD simulation. During the first 5 picoseconds of the equilibration phase the atoms coordinating Zn(II), according to the MM simulation were restraint to the ion via harmonic bonds. In the course of this pre-equilibration process that was conducted at the target temperature of 300K, the force constants of the bonds were lowered from 500 kcal/mol/Å^2^ to zero in 3 steps (250 kcal/mol/Å^2^, 100 kcal/mol/Å^2^, 0 kcal/mol/Å^2^). This was followed by 10 ps of equilibration and a 80 ps sampling phase. Since the core simulation, in contrast to the MM simulation, suggested robotnikinin forming additional hydrogen bonds with two histidine sidechains (H134 and H135) not described by QM, an additional simulation, including those residues into the QM zone has been set up in order to confirm the existence of these interactions. The starting structure for this second simulation was taken from the core simulation.

**Figure 2:**
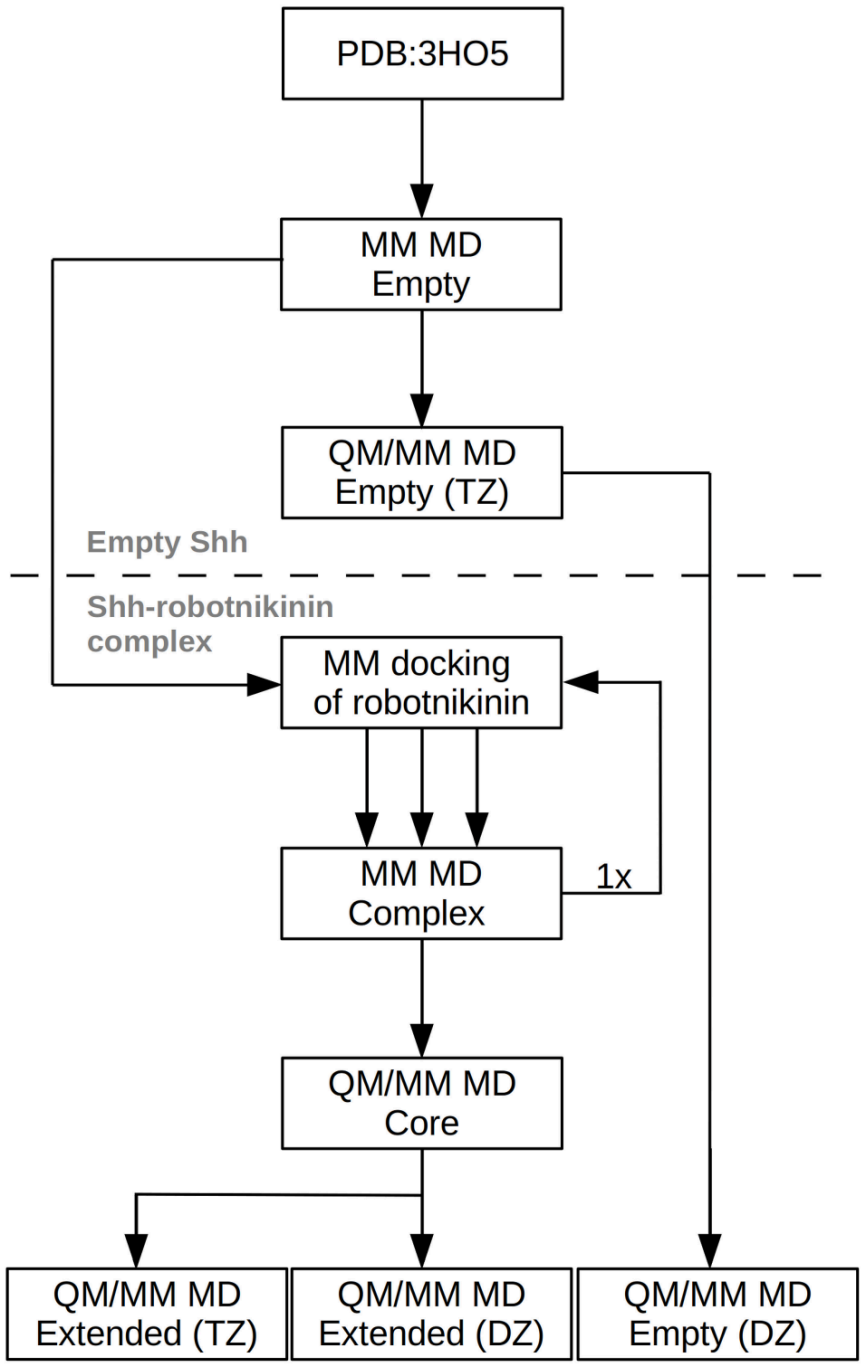
Chart depicting the process flow of the investigation. “Empty” refers to Shh without the ligand, TZ and DZ to QM/MM simulations utilizing triple or double zeta basis sets.

However, this enlargement of the system results in a significant increase of the computing time by a factor of approximately 2, thus a third simulation utilizing only a double zeta (DZ) basis has been conducted, reducing the time needed per simulation step by a factor of four. This simulation was used to gather additional configurations in order to improve the statistics on which the evaluated importance of the new found interactions are based. After a 10 ps equilibration phase, a 90 ps evaluation trajectory was sampled for this simulation. In order to assess the accuracy of the results obtained at the DZ level, a further simulation, this time of empty Shh was conducted. The starting point of this simulation was the equilibrated TZ simulation of empty Shh taken from the previously published study (Hitzenberger and Hofer, 2016). All settings remained the same, however the basis set was switched to the double zeta variant and the link bond parameters were adjusted accordingly. After 20 ps of equilibration, a 120 ps long sampling trajectory was produced. The process flow used in this investigation is visualized in Figure 2.

Calculation of MM forces, the QM/MM coupling and the MD simulation itself were all handled by the in-house-developed QMCF (Rode et al., 2006; Hofer et al., 2010) simulation package. All quantum mechanical calculations were carried out using TURBOMOLE (Turbomole, 2007) and the temperature was controlled by the Berendsen thermostat (Berendsen et al., 1984) with a relaxation time of 1.0 ps. The nonbonded interactions were calculated explicitely up to a distance of 10 Å, while long range interactions were dealt with by the reaction field method (Barker and Watts, 1973) assuming a permittivity of ε = 78.355. In order to allow for a time step of 2.0 fs, the SHAKE algorithm (Ryckaert et al., 1977) was applied and the equations of motion were solved using the velocity-Verlet integrator (Swope et al., 1982).

## 3. Results and discussion

### 3.1. MM MD simulations

**Figure 3:**
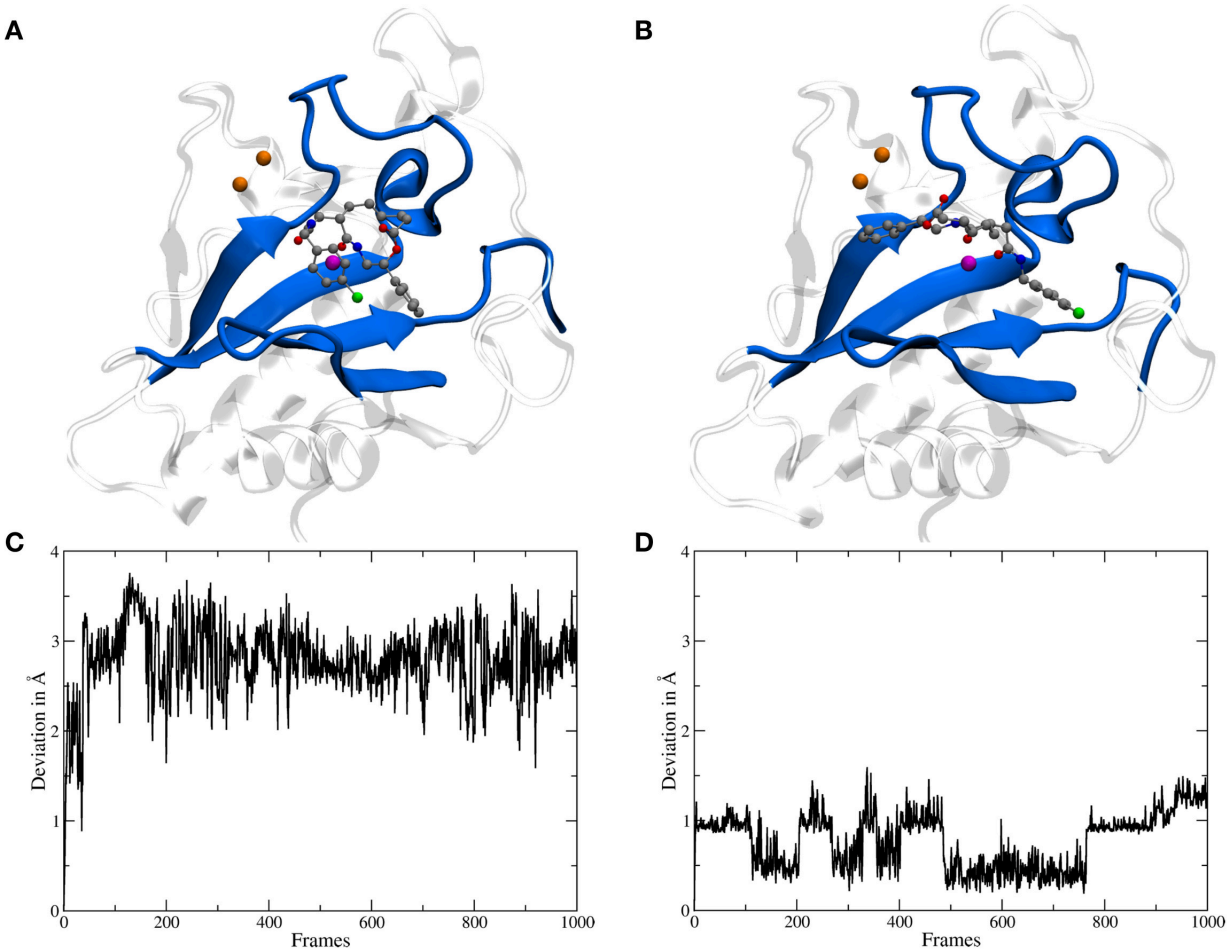
**(A)** A snapshot from an MM MD simulation started from a docking pose with the chlorine atom of the ligand pointing toward the Ca(II) ions (depicted in orange). The snapshot shows a configuration in which the chlorophenyl ring left the binding groove and is oriented toward the ligand's second aromatic ring instead of the Ca(II) ions. This is caused by a lack of stabilizing hydrophobic interactions with the protein as well as steric clashes and indicates that the chosen starting structure is not a stable binding mode. **(B)** A snapshot from an MM simulation started from the preferred docking pose. **(C)** Heavy atom RMSD of robotnikinin, calculated from the trajectory of the simulation shown in **(A)**. **(D)** Heavy atom RMSD of robotnikinin, derived from the trajectory of the simulation shown in **(B)**. Each frame in the RMSD plots represents 100 ps.

The binding site of Shh is located at the surface and shaped like a groove, therefore for a molecule like robotnikinin two general categories of binding poses are conceivable: one, where the chlorine atom points toward the region binding the Ca(II) ions and a second one, rotated by roughly 180° with the chlorine atom oriented in the opposite direction (see Figures 3B,C). During the docking phase two crucial properties were highlighted: Firstly, poses where an oxygen atom of robotnikinin coordinates to the Zn(II) ion were scored much higher than poses without direct robotnikinin-Zn(II) interactions. Nevertheless, MM MD simulations of complexes without direct Zn(II)-robotnikinin interactions have been conducted—all resulting in the disassociation of the complex. This indicates that the interaction with the Zn(II) site is very important for the stability of the complex which is not surprising, since interactions between metal ions and polar sites are much stronger than simple hydrogen bonds. Furthermore, from experimental studies of the Shh-Hhip complex, it is well established that the Zn(II) ion is indeed accessible to ligands (Bosanac et al., 2009). The second finding was that the category containing the poses with the chlorine pointing away from the Ca(II) site is by far the prevalent one since nearly every high ranked pose that could be taken into consideration for a simulation was of that variant. Very likely, sterical effects preventing the formation of stable hydrophobic interactions of the chlorophenyl ring with its environment play an important role. These sterical clashes occur because the part of the binding groove connecting the Zn(II) and the Ca(II) sites is slightly too short to cleanly incorporate the chlorophenyl ring, which is separated from the macrocycle by 4 covalent bonds. In contrast, the phenyl ring is directly bonded to the macrocylce and thus can be easily positioned in this part of the binding site. Nevertheless, in order to make sure that the most likely binding pose is selected for the subsequent QM/MM MD simulations, the highest ranked docking structures of both robotnikinin binding pose families have been selected and MM MD simulations have been performed following the protocol from the previous section. The chlorophenyl moiety did not interact very strongly with any part of the protein when oriented in the direction of the Ca(II) ions. Instead, it was switching positions very frequently, thus leading to many configurations in which the aromatic residues of the ligand interacted with each other occasionally leading to robotnikinin dissociating from the protein. The only stable simulation of this binding mode category resulted in a robotnikinin heavy atom root mean square deviation (RMSD) shown in Figure 3C, which is very high compared to the RMSD derived from the simulation in which the ligand is rotated, as shown in Figure 3D. From this and a visual inspection of the trajectories it can be gathered that the poses from the type depicted in Figure 3B seem to be the prevalent ones and therefore all following simulations were based on them.

**Figure 4:**
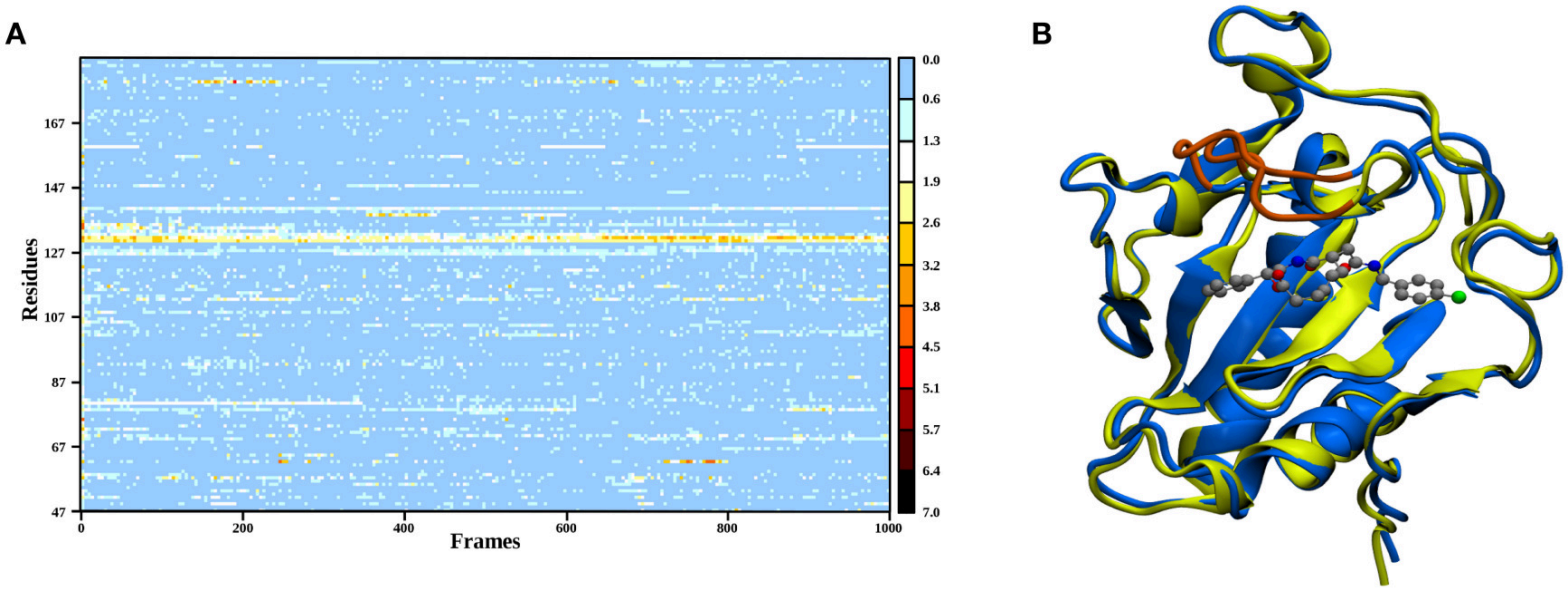
**(A)** Heatmap depicting the per-residue RMSDs of the classical simulations of loaded compared to empty Shh. The yellow and red regions indicate a high deviation. Note that the plot actually depicts the difference between the per-residue RMSDs of loaded versus empty Shh and the per-residue RMSDs derived from empty Shh versus itself, in order to emphasize only on the differences between the two systems. **(B)** Superposition of snapshots taken from loaded (blue) and empty (yellow) Shh simulations. The coil region spanning residues 131–136 showing the largest deviations is colored in orange.

**Figure 5:**
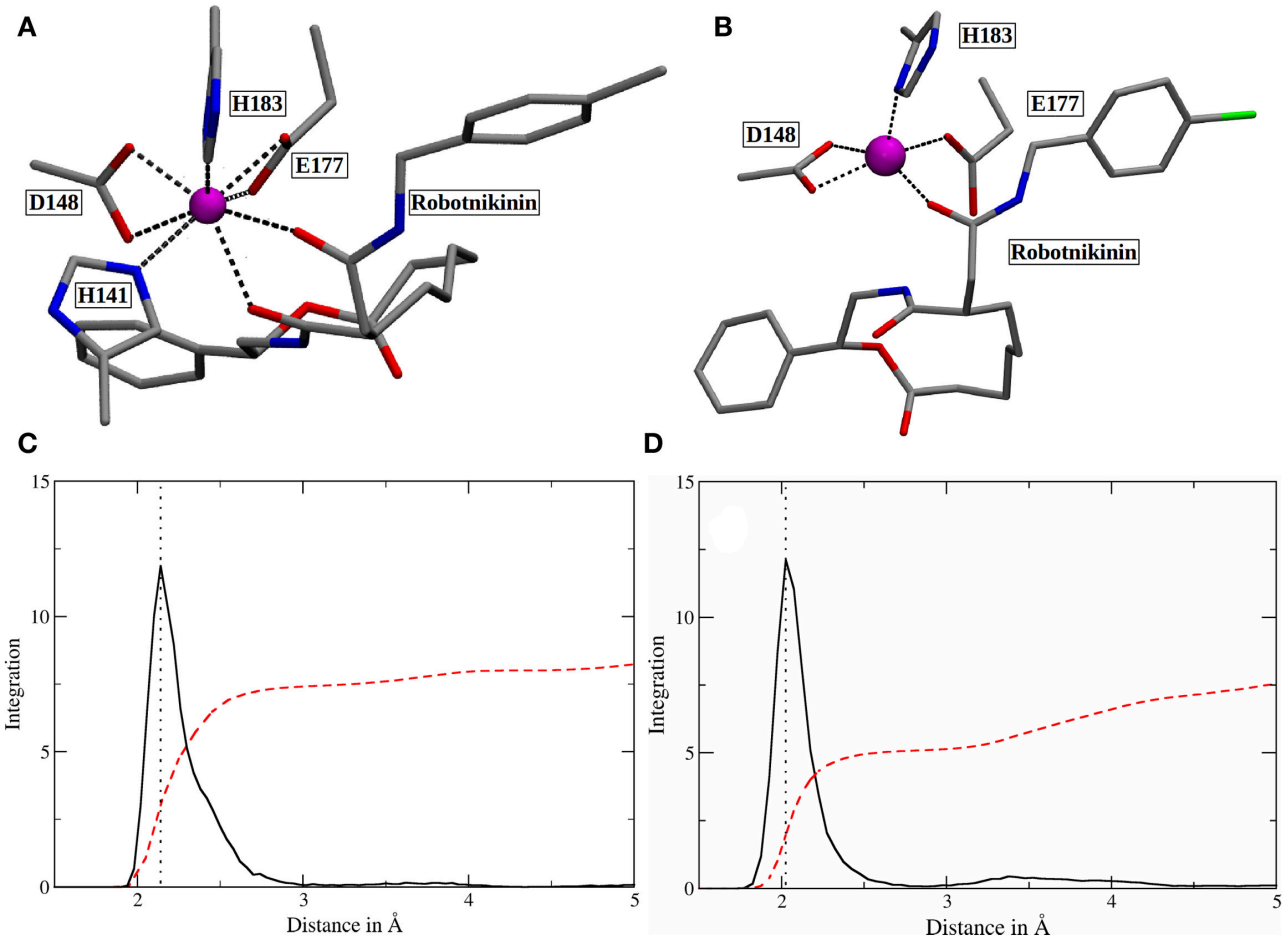
**(A)** Snapshot of the classical simulation, depicting 8-fold coordinated Zn(II) (magenta). **(B)** A representative snapshot from the core QM/MM simulation, showing the predominant coordination polyhedron around Zn(II). **(C,D)** Zn(II)-ligand RDFs of the classical **(C)** and the QM/MM simulation **(D)**. The RDFs are depicted in solid, black lines and their respective integrals in red. All oxygen and nitrogen atoms were considered for this plot. The dotted lines denote the peaks of the RDFs at 2.14 (MM) and 2.03 Å (QM/MM). Note that the y-axis labels refer to the integration only as the ligand atom densities are in arbitrary units.

In order to elucidate the structural differences between loaded and empty Shh, heavy-atom RMSDs for all individual residues comparing each evaluation frame of loaded Shh (MM MD) to the first frame of a simulation of empty Shh (Hitzenberger and Hofer, 2016) (also MM MD) have been calculated. To eliminate high RMSDs stemming from natural residue fluctuations, per-residue RMSDs of empty Shh versus itself have been calculated as well. These “natural fluctuations” have then been subtracted from the raw data. The resulting “corrected,” color coded per-residue RMSDs are shown in a heatmap plot in Figure 4A, which was generated using the “Heat Mapper” tool provided with the VMD (Humphrey et al., 1996) package. The regions showing a high deviation correspond to the coil region depicted in Figure 4B, functioning as a lid for the binding groove. The radial distribution function (RDF) calculated from the 100 ns MM MD simulation trajectory shows a mean coordination number of 7.5 oxygen or nitrogen atoms around the Zn(II) ion (counting up to a Zn(II)-ligand distance of 3.18 Å where the RDF shows a minimum). The ion is coordinated by H141, D148 (bidentate), E177 (mono- or bidentate), H183 and the amide-oxygen atoms of robotnikinin. The amide function in the macrocycle shows the higher affinity and is coordinated throughout the whole simulation (see Figure 5). The aromatic phenyl and chlorophenyl rings interact with the nearby sidechains of T126, H181, and Y175 via hydrophobic interactions. There seem to be no interactions between the ester or amide groups of robotnikinin with any of the surrounding sidechains. Instead, the used classical model predicts the formation of a hydrogen bond between the ester function and the macrocyclic amide. If a hydrogen-donor distance of under 3.0 Å and a donor-acceptor-hydrogen angle lower than 35° are used as geometrical criteria for a hydrogen bond, then 66.1% of the 1,000 configurations chosen for evaluation feature an intramolecular hydrogen bond.

### 3.2. QM/MM MD simulations

The core QM/MM MD simulation for which an 80 ps long evaluation trajectory has been sampled paints a different picture than the purely classical approach: Here, the Zn(II) ion is predominantly coordinated by D148 (bidentate), E177 (monodentate), H183 and the amide oxygen, part of the chain connecting the chlorophenyl ring of robotnikinin to its macrocycle. The coordination polyhedron is of quadratic pyramidal shape as can be seen in Figure 5B and according to an RDF (Figure 5D) that was calculated for the 4,000 frames of the evaluation trajectory, the mean coordination number is 5.1, when calculated up to a Zn(II) ligand distance of 2.83 Å (where the RDF reaches its first minimum), with the most likely ion-ligand distance being 2.03 Å. Incidentally, H141, part of the coordination sphere in empty Shh (Hall et al., 1995; McLellan et al., 2008; Bishop et al., 2009; Bosanac et al., 2009; Hitzenberger and Hofer, 2016) is mostly situated at distances between 3 and 4 Å of the ion, making way for robotnikinin, E177 and enabling a bidentate binding mode of D148. However, there are also configurations where it directly binds the ion thereby creating a quadratic bipyramidal coordination polyhedron. Another interesting aspect of the coordination site around the ion is E177, which has been shown to be mostly bridged by a water molecule in studies of empty Shh (Hitzenberger and Hofer, 2016) or X-ray derived crystal structures of Shh bound to Hhip (McLellan et al., 2008; Bishop et al., 2009; Bosanac et al., 2009). However, the conducted QM/MM MD simulation predicts direct E177-Zn(II) coordination surely aided by the MM derived starting structure also predicting such a coordination. In the previous study of empty Shh (Hitzenberger and Hofer, 2016), the MM MD trajectory used as starting point for the QM/MM simulation erroneously predicted E177 to directly interact with the ion during the entire simulation. However, the QM/MM model switched to the experimentally predicted coordination sphere almost instantly after heating the system. This was not the case in this QM/MM simulation, however, in the present case E177-Zn(II) binding is not implausible because the second oxygen atom of E177 forms a very strong hydrogen bond with the amide function in the macrocycle of Shh, thereby also directing the amide oxygen atom toward histidines H134 and H135 enabling potential hydrogen bonding. Therefore, the presence of E177 increases the number of possible protein-ligand interactions thus stabilizing the Sonic Hedgehog-robotnikinin complex. Although the continued presence of a water molecule in the binding site would be possible, it is however unlikely that it would aid the binding of robotnikinin as strongly as E177 does because a water molecule is neutral and possesses just one atom that can function as an electron donor, whereas E177 bridges two positively charged sites via its two spatially separated oxygen atoms. Furthermore, the elimination of strongly bound water molecules aids to ligand binding via a favorable entropy contribution (Ladbury, 1996; Li and Lazaridis, 2005; Huggins, 2015). In the previously conducted QM/MM simulation it has also been shown that while the bridged binding of E177 to Zn(II) is absolutely predominant, there are also configurations where it binds directly (Hitzenberger and Hofer, 2016), suggesting that this form of coordination is not entirely unfeasible also without the presence of a ligand. Upon closer investigation of the E177-robotnikinin interaction it becomes clear that it is a very stable one, as the mean distance between the E177 oxygen and the hydrogen atom of the amide is 2.29 Å and applying the same H-bond criteria as before, 85.7% of all sampled configurations exhibit this particular hydrogen bond. Another argument for the exclusion of water molecules around the metal ion center is the fact that binding of robotnikinin to Shh renders Zn(II) practically inaccessible to the solvent. This is illustrated by the Zn(II)-water oxygen RDF of the core simulation: The first peak can be found at approx. 6.6 Å where integration up to this point yields 1.5 water molecules. At a separation of 5 Å integration of the RDF indicates a mean number of just 0.006 water molecules. This suggests that it is very unlikely that water re-enters the active site once it has left. If all these features are taken into consideration, it seems very plausible that robotnikinin binds to Zn(II) via a direct ionic bond, as well as indirectly via the residue E177.

The mean distances of residues H134 and H135 to the macrocyclic amide group of robotnikinin in the core QM/MM investigation are 4.41 and 2.53 Å, respectively and additionally there are some close contacts between H134 and the macrocyclic ester group (with an average distance of 4.09 Å), hinting at the existence of favorable interactions between these histidines and the ligand. Calculating the RMSDs for just the sidechains (considering only heavy atoms) of these two residues results in a mean deviation of 0.481 Å, which compared to the RMSDs derived from the MM simulation (1.187 Å) or all sidechains in the QM/MM described protein (0.874 Å) is a very low value, further suggesting the presence of stabilizing interactions. However, if the same hydrogen bond criteria used for the MM simulations are taken into account, then in only 19.1% of all the frames H135-amide hydrogen bonds are present, while 4.8% of all sampled configurations show H134-amide H-bonding and only 43 out of 4,000 configurations fulfill the criteria for accepting the existence of a hydrogen bond between H134 and the ester functionality. Apparently, while the electrostatic interactions between the histidines and robotnikinin seem to be rather strong, the angle between donor, hydrogen atom and acceptor deviates quite far from 180° most of the time. One of the likely reasons for this behavior is the fact that the histidines in question are not part of the QM zone of this simulation, therefore all observed interactions between them and robotnikinin are somewhat error prone and thus should be closer observed by including H134 and H135 in the QM region. However, as QM calculations scale very unfavorably with system size, beside a larger triple zeta (TZ) simulation, the system was also simulated utilizing only a double zeta basis set in order to attain a larger number of configurations and thus gain data that is statistically more conclusive.

**Figure 6:**
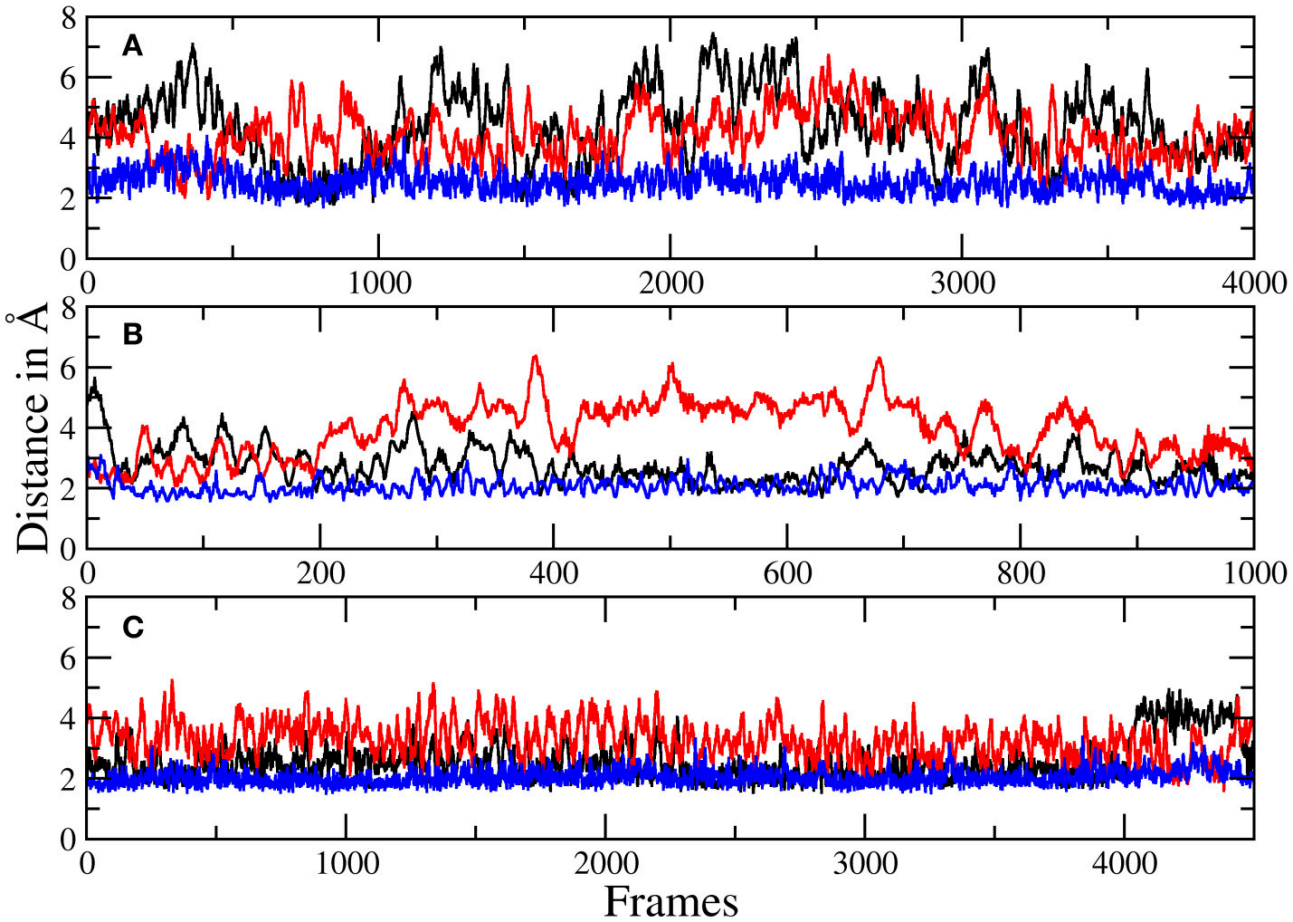
Plots of the distances between H134 and robotnikinin's macrocyclic amide group (black), ester (red), as well as the separation of H135 and the macrocyclic amide function (blue) of robotnikinin. Each frame represents a time span of 0.02 ps. **(A)** Core simulation. **(B)** Extended simulation (TZ). **(C)** Extended simulation (DZ).

**Figure 7:**
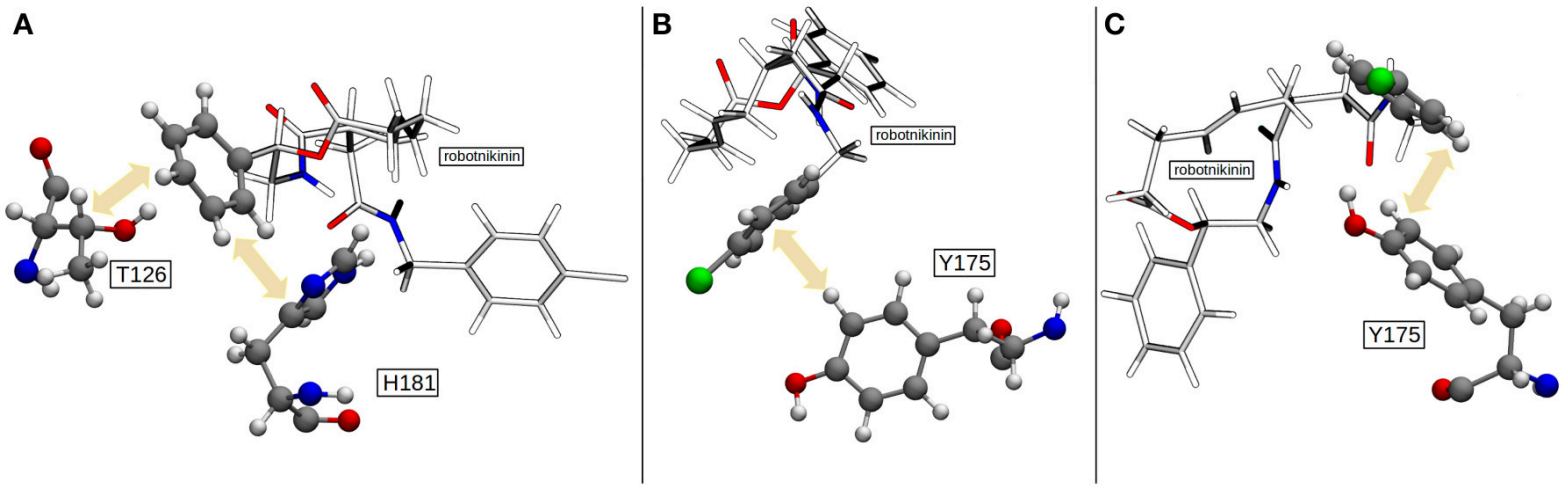
**(A)** The most common geometry of the hydrophobic interactions between T126, H181, and robotnikinin. **(B)** A configuration displaying T-shaped π–π stacking of the aromatics belonging to robotnikinin and Y175, respectively. **(C)** The more frequent parallel-displaced stacking geometry between the chlorophenyl ring of robotnikinin and Y175 as witnessed during the QM/MM simulations.

The more sophisticated TZ simulation suggests that the interaction between the histidines and the macrocylce of robotnikinin is more distinct than predicted by the core QM/MM simulation as the mean amid histidine distances are reduced to 2.77 Å (H134) and 2.08 Å (H135) in addition, also the H-bond acceptor oxygen of the ester is on average separated from the donor hydrogen of H134 by 3.94 Å and occasionally close enough for hydrogen bonding. The simulation predicts a hydrogen bond population of 80.7% for H135-amide, 40.6% for H134-amide and 1.4% for H134-ester, respectively. The mean distance between the closest E177 oxygen atom and the hydrogen of the macrocyclic amide is 2.14 Å, with a hydrogen bond abundance of 95.3%, implying the E177-robotnikinin and the H134/135-robotnikinin interactions promote each other by orienting the amide group in a favorable position. The statistically more robust (but less accurate) 90 ps DZ simulation predicts interactions that are even stronger than in the TZ case yet only by a small margin. The mean distances between H134, H135 and the amide are predicted to be 2.56 and 2.05 Å, respectively, with the average separation between H134 and the ester being 3.27 Å. Of the 4,500 sampled configurations, 87.5% show a hydrogen bond between H135 and the amide, 50.7% between H134 and the amide and 5.4% between H134 and the ester. The mean E177-amide distance is predicted to be 2.13 Å, with a hydrogen bond abundance of 92.9%. A summary of the discussed H-bond results regarding the interactions of H134, H135, and E177 with robotnikinin is provided in Table 3. To further highlight the binding motif, robotnikinin–sidechain distance plots are depicted in Figures 6A–C, taking only into consideration the atoms closest to each other, as these are regarded to be the mediators of the respective H-bonds. The interactions found for the aromatic rings are the same as in the MM MD simulation, which is unsurprising since in both cases they were described solely by the force field and the predominant stacking geometries are depicted in Figure 7.

**Table 3:** Distances (in Å) between residues H134, H135, E177 and the respective functional group of robotnikinin.

	**Simulation #1 Core**				**Simulation #2 Extended (TZ)**				**Simulation #3 Extended (DZ)**			
	**Amide**		**Ester**		**Amide**		**Ester**		**Amide**		**Ester**	
	**Dist**.	**Occ**.	**Dist**.	**Occ**.	**Dist**.	**Occ**.	**Dist**.	**Occ**.	**Dist**.	**Occ**.	**Dist**.	**Occ**.
H134	4.41	4.8	4.09	<0.1	**2.77**	**40.6**	**3.94**	**1.4**	**2.56**	**50.7**	**3.27**	**5.4**
H135	2.53	19.1	n.a.	n.a.	**2.08**	**80.7**	n.a.	n.a.	**2.05**	**87.5**	n.a.	n.a.
E177	**2.29**	**85.7**	n.a.	n.a.	**2.14**	**95.3**	n.a.	n.a.	**2.13**	**92.9**	n.a.	n.a.

*The occurrences of hydrogen bond formation are given in percent. Results stemming from purely QM described interactions are printed in bold font*.

**Figure 8:**
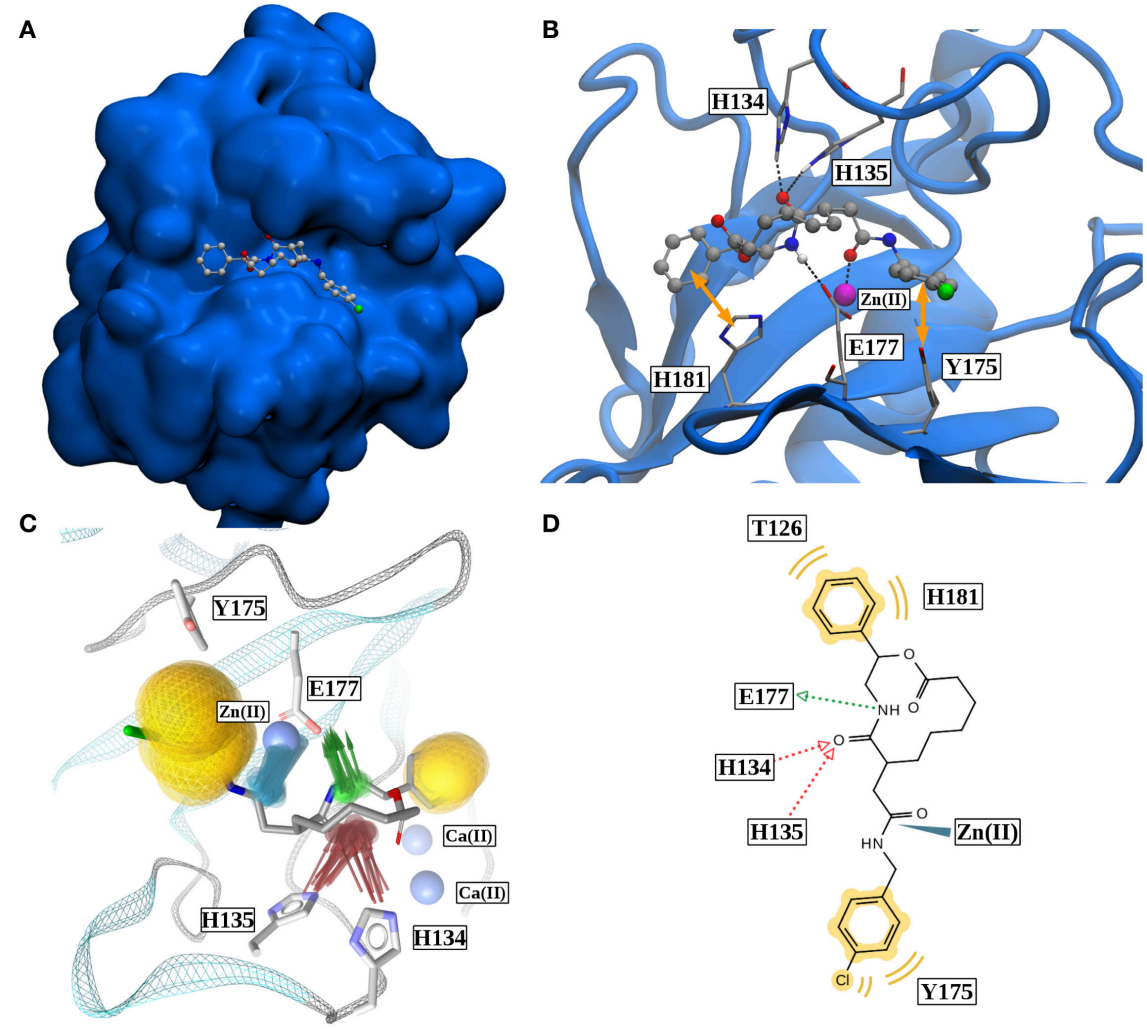
**(A)** Binding pose of robotnikinin in Sonic Hedgehog (van der Waals surface). **(B)** Most important interactions between robotnikinin and Sonic Hedgehog, depicted in a snapshot of the extended TZ simulation. The Zn(II) ion is colored in magenta, H-bonds and metal-ligand interactions are represented as black, dashed lines, while hydrophobic interactions are shown as yellow arrows. With the exception of hydrogen atoms that are part of H-bonds, only heavy atoms are shown. **(C)** Superposition of 20 interaction profiles constructed from every fiftieth frame of the extended TZ sampling trajectory. Green arrows represent H-bond donors, while acceptors are colored in red, hydrophobic interactions are shown in yellow and ligand-metal interactions are depicted in blue. **(D)** Two-dimensional interaction profile of robotnikinin with Shh, adapted from the output of LigandScout. The color-coding is analogous to the one used in **(C)**.

If all these findings are taken into consideration, one can conclude that, besides the very strong Zn(II)-robotnikinin ionic bond, the most stable interaction found by the QM/MM MD simulations is the hydrogen bond between E177 and the macrocyclic amide hydrogen as this interaction has an occurrence ranging from 85.7% (core QM/MM simulation) to 92.9 and 95.3% in case of the DZ and TZ extended QM/MM simulations. The second most important hydrogen bond exists between H135 and the macrocyclic amide of robotnikinin with an abundance close to 90% in both extended simulations including H135 and H134 in the QM zone. Another frequently occurring interaction was identified between H134 and the same amide, which is present in roughly half of all sampled configurations of the extended QM/MM MD simulations. Also predicted to exist but not nearly as important as the other interactions since only witnessed in 1 to 5% of all frames is an H-bond between the ester and H134. The binding pose of robotnikinin in Shh as predicted by the extended TZ simulation is shown in Figure 8A and respective interactions mediating this orientation are depicted in Figure 8B. In order to further confirm these findings and to construct a three-dimensional map of all important interactions between robotnikinin and Sonic Hedgehog every fiftieth configuration of the extended TZ sampling trajectory was employed to create twenty different interactions models, utilizing the software tool LigandScout (version 4.09) (Wober and Langer, 2005; Wieder et al., 2017). The resulting three- and two-dimensional interaction maps are shown in Figures 8C,D, confirming the observations from the distance and angle based trajectory analysis. Not shown in the pictures is residue K88, which is very close to robotnikinin's phenyl ring with a mean distance of 5.48 Å (core QM/MM simulation). It is conceivable that if the phenyl ring was modified with an additional negatively charged moiety then a hydrogen bond interaction with K88 could be achieved, further stabilizing the robotnikinin-Shh complex.

In order to assess how robust the predictions based on the DZ basis are, a QM/MM MD simulation of empty Shh was conducted, utilizing the DZ basis (along with adjusted link-bond parameters) but other than that the same settings as in the previous study (Hitzenberger and Hofer, 2016). The results suggest that with a predicted Zn(II) coordination number of 5.5 (calculated up to a distance of 2.78 Å), where the ion is commonly coordinated by E177, along with H141, D148, H183 and one or two water molecules, in contrast to the results found by the TZ simulation (CN of 4.4 and very rare direct E177-Zn(II) bonding), that a double zeta basis might not be sufficient to accurately describe such a system when applied to a simulation utilizing the BP86 functional. Nevertheless, the obtained results are still much more accurate than those of the purely classical simulation, however, it is not as close to the experimental data as the TZ simulation. For this reason, all structurally relevant data for this study were taken from the TZ investigations. However, as the DZ simulation is nearly four-times faster than the TZ case and the results regarding hydrogen bond population very close to the data taken from the TZ investigations they can be viewed as a statistically more robust confirmation of the behavior witnessed in the shorter TZ simulation. The calculation of the solvent accessible surface area (SASA) of robotnikinin from the 1,000 configurations sampled in the extended TZ simulation resulted in a mean value of 297.6 Å^2^ (±0.7 Å^2^, *P* = 95%, *SD* = 12.0 Å^2^, *n* = 1,000) which is very close to the 300.5 Å^2^ (± 1.4 Å^2^, *P* = 95%, *SD* = 22.7 Å^2^, *n* = 1,000), obtained from the classical simulation. This finding is unsurprising because no major configurational changes have been witnessed, instead the most notable differences between the QM/MM and MM structures concern areas which are buried in the Shh's binding groove in both cases.

For further reference, a representative PDB file containing the system (taken from the extended TZ simulation) is provided as Supplementary Material. In order to reduce the size of the file, all solvent molecules have been removed.

## 4. Conclusions

In the presented study, a reasonable binding mode for the small molecule inhibitor of Sonic Hedgehog, robotnikinin is suggested. The interactions, identified via a series of QM/MM MD simulations were sufficiently strong to stabilize the Shh-robotnikinin complex throughout the investigations and enabled a binding mode in which the ligand interacts with six amino acids and the Zn(II) ion present in the binding groove of Shh. The most important and stable interactions are the ionic bond between the Zn(II) ion of Sonic Hedgehog and one of the oxygen atoms of robotnikinin as well as a hydrogen bond between E177 and the macrocyclic amide group of robotnikinin, bridging the inhibitor with the ion. Other very important contributions include hydrogen bonds between H134/H135 and the macrocyclic amide group as well as hydrophobic interactions between the aromatic rings of the ligand and the sidechains of residues T126, Y175 and H181, predominantly forming π–π interactions.

In all conducted simulations K88, also known to bind complex partners of Shh (Bosanac et al., 2009), is sufficiently close to the phenyl ring of robotnikinin in order to form an H-bond if an electron donor function were present at a suitable position, thereby potentially further improving the affinity of Shh to the small molecule inhibitor and in addition preventing the contribution of K88 to the formation of complexes with other proteins.

Even though in the core simulation the QM/MM interface very likely prevented donor-hydrogen-acceptor geometries commonly regarded as required to confirm the existence of hydrogen bonds between the classically described histidines H134/H135 and the QM described ligand, their behavior (low RMSD, close proximity to the inhibitor) was as a very strong hint for the presence of important interactions between those molecules hence justifying the utilization of the applied embedding scheme. It is, however, still very important to stress that in order to obtain reasonable structural data a careful choice of the QM zone is necessary making sure that all potential ligand protein interactions are treated at the same level of theory. However, as QM calculations scale very unfavorably with the number of atoms in the system, the size of the QM region has to be as small as possible in order to attain a sufficient number of configurations from which statistically robust data can be derived. Therefore, a reasonable compromise between method accuracy and system size is very important to sample a sufficient number of configurations that are also physically meaningful. In the presented work this could be achieved by conducting three different QM/MM simulations of the same system, differentiated by their respective basis set and system sizes.

The purely classical model of the studied system, on the other hand, predicts a vastly over-coordinated Zn(II) site due to an overestimation of the ion-ligand interaction strength, which is probably also the main reason for the absence of the hydrogen bonds between robotnikinin and E177 or H134 and H135 (as they seem to promote each other, presumably due to the partial double bond character of the C-N bond in the amide). This can be viewed as a further indication that a description of the system based solely on force fields of the kind as employed in this study are not ideally suited for the physically correct description of systems involving transition metal ions. Overall, this work also highlights the capabilities of an iterative docking/(QM)MM MD cycle if used to improve the prediction power of *in silico* ligand-receptor binding studies.

## Author contributions

All simulations were executed and evaluated by MH. The simulations were performed using code written and implemented by TH who also contributed to the conceptualization of the investigation. The manuscript was drafted by MH and revised by TH and DS who was consulted due to her expertise in protein ligand docking.

### Conflict of interest statement

The authors declare that the research was conducted in the absence of any commercial or financial relationships that could be construed as a potential conflict of interest.
